# Defining the Genes Required for Survival of Mycobacterium bovis in the Bovine Host Offers Novel Insights into the Genetic Basis of Survival of Pathogenic Mycobacteria

**DOI:** 10.1128/mbio.00672-22

**Published:** 2022-07-14

**Authors:** Amanda J. Gibson, Jennifer Stiens, Ian J. Passmore, Valwynne Faulkner, Josephous Miculob, Sam Willcocks, Michael Coad, Stefan Berg, Dirk Werling, Brendan W. Wren, Irene Nobeli, Bernardo Villarreal-Ramos, Sharon L. Kendall

**Affiliations:** a Centre for Emerging, Endemic and Exotic Diseases, Pathobiology and Population Sciences, Royal Veterinary College, Hatfield, United Kingdom; b Institute of Structural and Molecular Biology, Biological Sciences, Birkbeck, University of London, London, United Kingdom; c London School of Hygiene and Tropical Medicine, London, United Kingdom; d Animal and Plant Health Agency, Addlestone, Surrey, United Kingdom; Weill Cornell Medical College

**Keywords:** Tn-seq, gene essentiality, One Health, tuberculosis, virulence factors

## Abstract

Tuberculosis has severe impacts on both humans and animals. Understanding the genetic basis of survival of both Mycobacterium tuberculosis, the human-adapted species, and Mycobacterium bovis, the animal-adapted species, is crucial to deciphering the biology of both pathogens. There are several studies that identify the genes required for survival of M. tuberculosis *in vivo* using mouse models; however, there are currently no studies probing the genetic basis of survival of M. bovis *in vivo.* In this study, we utilize transposon insertion sequencing in M. bovis AF2122/97 to determine the genes required for survival in cattle. We identify genes encoding established mycobacterial virulence functions such as the ESX-1 secretion system, phthiocerol dimycocerosate (PDIM) synthesis, mycobactin synthesis, and cholesterol catabolism that are required *in vivo*. We show that, as in M. tuberculosis H37Rv, *phoPR* is required by M. bovis AF2122/97 *in vivo* despite the known defect in signaling through this system. Comparison to studies performed in species that are able to use carbohydrates as an energy source, such as M. bovis BCG and M. tuberculosis, suggests that there are differences in the requirement for genes involved in cholesterol import (*mce4* operon) and oxidation (*hsd*). We report a good correlation with existing mycobacterial virulence functions but also find several novel virulence factors, including genes involved in protein mannosylation, aspartate metabolism, and glycerol-phosphate metabolism. These findings further extend our knowledge of the genetic basis of survival *in vivo* in bacteria that cause tuberculosis and provide insight for the development of novel diagnostics and therapeutics.

## INTRODUCTION

Bacteria belonging to the Mycobacterium tuberculosis complex (MTBC) have devastating impacts on both animal and human populations. Mycobacterium bovis, an animal-adapted member of the MTBC and one of the main causative agents of bovine tuberculosis (bTB), remains endemic in some high-income settings despite the implementation of a test-and-slaughter policy. In low- and middle-income settings, the presence of bTB in livestock combined with the absence of rigorous control measures contributes to the risk of zoonotic transmission (1, 2). Control measures based on cattle vaccination utilize the live attenuated vaccine M. bovis BCG, but the efficacy of this vaccine remains low in field situations (3, 4). In addition to vaccines, the development of diagnostic tools for the identification of infected individuals is crucial for the management of transmission. Vaccination with M. bovis BCG sensitizes animals to the diagnostic tuberculin skin test; therefore, sensitive and specific differentiating diagnostic strategies are a current imperative (5, 6).

The increased accessibility of whole-genome fitness screens has allowed the assessment of the impacts of the loss of gene function on bacterial survival (7). Such screens have been invaluable in identifying novel drug targets or candidates for the generation of new live attenuated vaccines in a number of bacterial pathogens, including M. tuberculosis (8–13). Studies utilizing whole-genome transposon mutagenesis to examine gene fitness *in vivo* in M. tuberculosis H37Rv have been limited to mouse models (8–10). These models do not faithfully replicate the granulomatous pathology associated with TB, nor do mice contain the same repertoire of CD1 molecules expressed by bovine T cells required to present mycobacterial lipid antigens (14). Whole-genome transposon mutagenesis screens utilizing nonhuman primates are limited because screening is restricted to smaller mutant pools (15). To date, transposon insertion sequencing (Tn-seq)-based studies in the context of bTB in cattle have only been performed using M. bovis BCG strains (16, 17).

In this study, we use Tn-seq to determine the genes required for survival of M. bovis AF2122/97 directly in cattle. We show that genes involved in the biosynthesis of phthiocerol dimycocerosates (PDIMs), the ESX-1 secretion system, cholesterol catabolism, and mycobactin biosynthesis are essential for survival in cattle, corroborating current knowledge of gene essentiality in members of the MTBC (8–10, 16, 17). We identify differences in the requirement for genes involved in cholesterol transport and oxidation in the fully virulent M. bovis AF2122/97 strain. We also identify several novel genes required for survival *in vivo* that have not been previously described in members of the MTBC.

## RESULTS AND DISCUSSION

### Generation and sequencing of the input library.

We generated a transposon library in M. bovis AF2122/97 using the phagemid system as previously described (18, 19). Sequencing of the input library showed that transposon insertions were evenly distributed around the genome, and 27,419 of the permissible 66,931 thymine-adenine dinucleotide (TA) sites contained an insertion representing an insertion density of ~41% (see input library Fig. S1 and Table S1 at https://doi.org/10.5281/zenodo.6598446). The M. bovis AF2122/97 genome has 3,989 coding sequences, and insertions were obtained in 3,319 of these; therefore, the input library contained insertions in 83% of the total coding sequences.

### Mycobacterium bovis-specific immune responses were observed in cattle.

**FIG 1:**
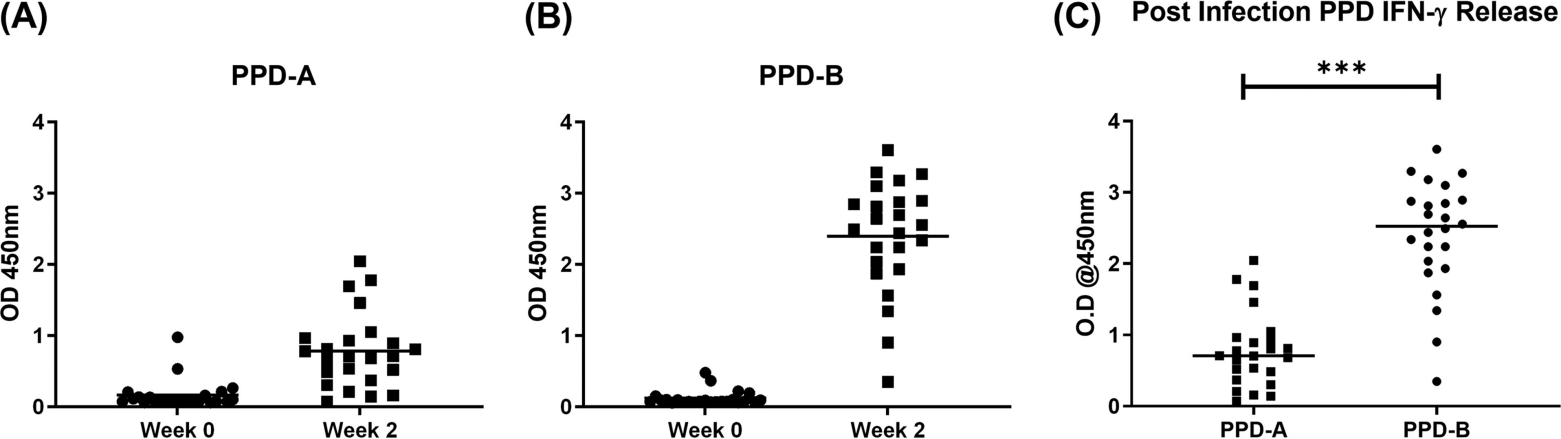
bTB-specific IFN gamma release in cattle infected with the M. bovis Tn library. (A and B) Blood was collected from all 24 animals on the day of infection and 2 weeks later. No response was detected to either PPD-A or PPD-B antigen stimulation prior to infection (week 0). (C) All animals presented a significant and specific response to PPD-B compared to PPD-A as determined by a paired *t* test using GraphPad Prism. ***, *P* ≤ 0.001.

Twenty-four clinically healthy calves of approximately 6 months of age were inoculated with the library through the endobronchial route. Infection was monitored by interferon gamma (IFN-γ) release assay (IGRA) at the time of inoculation and 2 weeks postinfection. M. bovis-specific immune responses were observed for all study animals at 2 weeks postinfection (Fig. 1A and B). Each animal presented a very low background of circulating IFN-γ together with a statistically significant (***, *P* ≤ 0.001) increase in IFN-γ release in response to purified protein derivative from M. bovis (PPD-B) compared to PPD from M. avium (PPD-A) antigens (Fig. 1C). This indicates that infection with the library was successfully established in the cattle.

### Pathology associated with infection was greater in the lung and thoracic lymph nodes.

**FIG 2:**
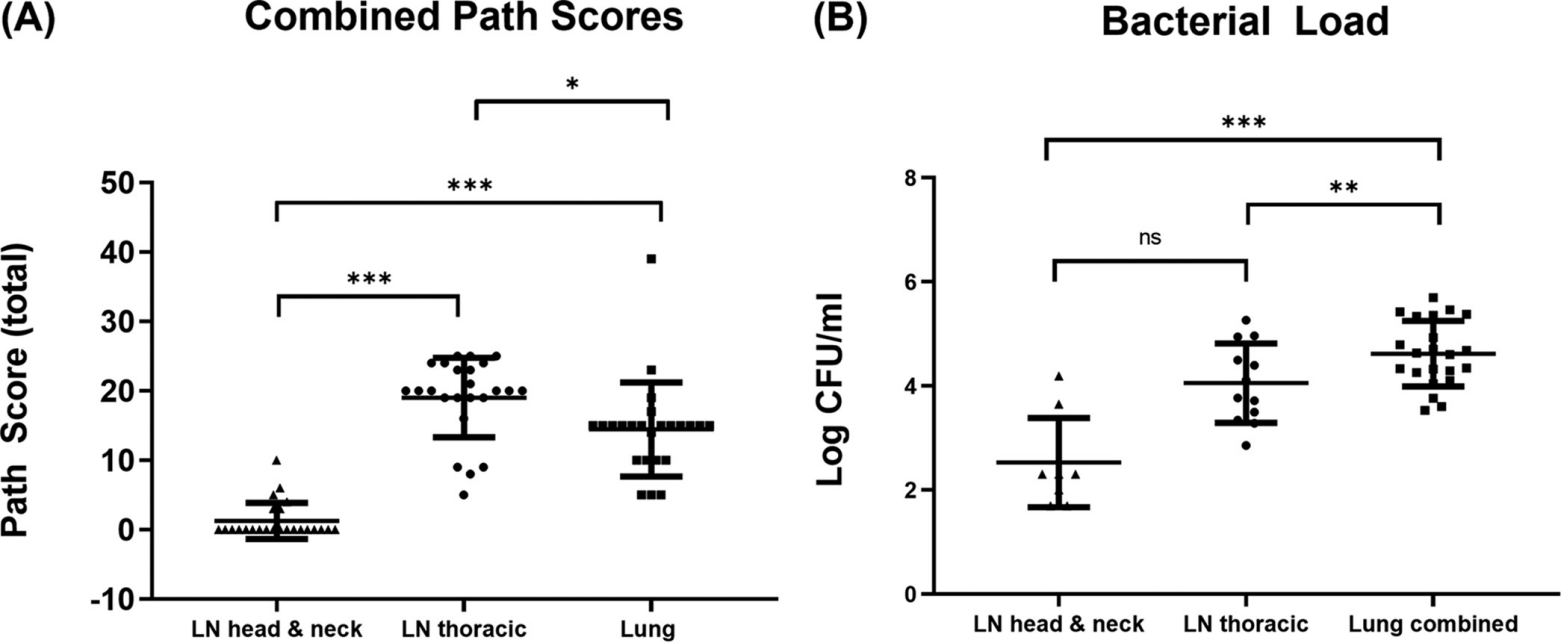
Tissue pathology and bacterial load in tissue sites. (A) Six weeks after infection, animals were subjected to postmortem examination. Gross pathology and evidence of TB-like granulomas lesions were scored. Data presented are the mean across animals of the total scores for each tissue group from 24 animals ± the standard deviation. Lung and thoracic lymph nodes were observed to contain the highest pathology compared to head and neck lymph nodes. (B) For bacterial load estimation, aliquots of macerates were plated onto modified 7H11 agar containing kanamycin. Colonies were counted after 3 to 4 weeks growth. Data are presented as mean log_10_ CFU/mL per collected tissue group ± standard deviation. Lung tissue contained the highest bacterial burden compared to thoracic and head and neck lymph nodes as determined by one-way analysis of variance (ANOVA) using GraphPad Prism. *, *P* = 0.01; **, *P* = 0.002; ***, *P* ≤ 0.001.

Animals were culled at 6 weeks postinfection. Lung sections and upper (head and neck) and lower (thoracic) respiratory tract-associated lymph nodes were examined for gross lesions. Lesions typical of M. bovis infection were observed in the tissues examined (see Table S2). Pathology scores are shown in Fig. 2A. Greater pathology was observed in the lung and thoracic lymph nodes than in the head and neck lymph nodes.

### Higher bacterial loads were associated with the lung and thoracic lymph nodes.

Bacterial counts were highest in lesions derived from the lung compared to those from the thoracic lymph nodes and head and neck lymph nodes (Fig. 2B). The lowest bacterial counts were observed within the head and neck lymph nodes. However, this was not significant compared to thoracic lymph nodes. The volume of each macerate varied depending on lesion size. Considering macerate volume, average bacterial loads of 10^7^, 10^6^, and 10^5^ were recovered from lesions from samples of the lungs, thoracic lymph nodes, and head and neck lymph nodes, respectively.

### Recovery and sequencing of *in vivo*-selected transposon libraries.

In order to recover the Tn library from harvested tissue, ~10^5^ to 10^6^ CFU from lungs and thoracic lymph nodes were plated. Samples from 4 cattle were lost due to fungal contamination; therefore, samples processed represent 20 cattle. Lung samples were plated from all 20 animals, and thoracic lymph nodes samples were plated from 6 cattle. Bacteria were grown for 4 to 6 weeks before harvesting for genomic DNA extraction and sequencing (see Table S1 for assignation of sequencing files). The insertion densities of the output libraries were compared to the input library for each sample (Fig. S2). Libraries recovered from lung lesions from 20 different cattle contained an average of 14,456 unique mutants, and those recovered from the thoracic lymph nodes contained an average of 16,210 unique mutants. Given that the input library contained 27,419 unique mutants, this meant that there was an ~40 to 50% reduction in insertion density in the output libraries compared with the input. Good coverage of coding sequences (CDSs) was maintained, as the output libraries still contained insertions in (on average) 68 to 70% of the open reading frames. Given the loss of diversity of the individual output libraries, we pooled samples from the lungs and separately from the lymph nodes. The insertion densities of the pooled samples from the lungs were 33,039/66,931 permissible TA sites, and from the nodes were 25,072/66,931 permissible TA sites. This represented ~50% and ~38% saturation. Therefore, using this approach, the diversity of the input pools was maintained.

Calculation of the log_2_ fold change in the read counts between the input and output libraries allowed a measurement of the impact of the insertions on the survival of mutants in cattle. In order to determine statistically significant changes in the representation of mutants between the input and output libraries, we analyzed pooled samples from the lungs (20 cattle) and pooled samples from the lymph nodes (6 cattle). However, in recognition that cattle are genetically more heterogenous than standard laboratory mice, we have included data where we have calculated the log_2_ fold change between the output library and the input library for individual samples in addition to the pooled data set. The entire data set is shown in Table S3, and a volcano plot is shown in Fig. S3.

A comparison of the mean log_2_ fold change between lung and lymph node samples showed good correlation (Spearman’s rho, 0.88; *P* < 2.2e-16) (Fig. S4). TRANSIT resampling was performed to compare the composition of the mutant population in the lungs and thoracic lymph nodes of paired cattle; it was also applied to compare all the thoracic lymph nodes with the lungs of all cattle samples. TRANSIT analysis did not find any statistically significant differences, indicating that there were no differences in mutant composition between the tissue sites.

Using an adjusted *P* value cutoff of ≤0.05 and a log_2_ fold change of −1.5 in either lungs or lymph node, insertions in 300 genes caused significant attenuation in cattle. Of these genes, 220 had been previously described as being required *in vivo* in M. tuberculosis H37Rv in standard mouse models through the use of whole-genome Tn screens representing ~73% overlap with the previous literature (8–10). These genes are given in Table S3 (“Significant genes” tab). No insertion mutants were significantly over-represented in the library. Although *Mb0025* was over-represented in both lungs and nodes (log_2_ fold change, 7 to 8 in the pooled analyses), significant cutoffs were not reached, and this may be reflective of a lower number of TA sites in this gene, which limits statistical power. *Mb0025* overlaps with *Mb0024* and is the result of a frameshift mutation in the AF2122/97 genome. This mutation is also found in other assembled M. bovis genomes, and we could find no evidence for lack of conservation of this frameshift mutation in global collections (20). *Mb0024* and *Mb0025* represent orthologs of the 5′ and 3′ ends of *Rv0024*, respectively, which is annotated as a p60 homologue involved in cell-to-cell spread (21). The functionality of *Mb0024* and *Mb0025*, or the impact on the transposon insertion, is not known.

### Comparison with mutations known to cause attenuation in the MTBC.

**FIG 3:**
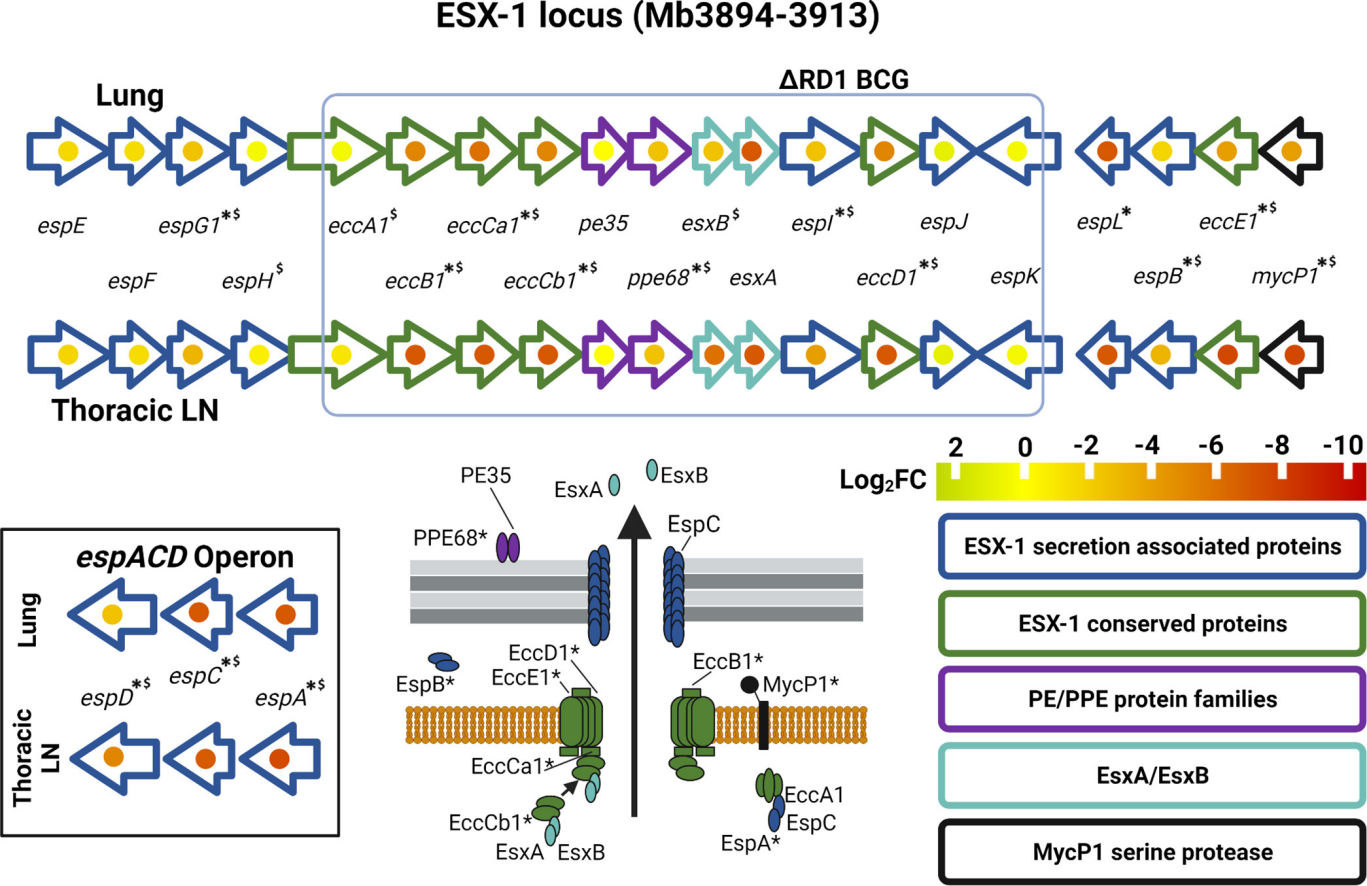
Fold changes caused by transposon insertions in the ESX-1 secretion system in the lungs and lymph nodes of infected cattle. Asterisks indicate that genes were significantly attenuated in pooled output samples (adjusted *P* ≤ 0.05 in either lungs or node). A dollar sign indicates those genes that are attenuated in H37Rv in mice models. The genes are grouped according to function as indicated by the color scheme. The log_2_ fold changes are indicated on a yellow-to-red scale and presented as a dot in the center of the gene.

Insertions in the RD1-encoded ESX-1 type VII secretion system secreting virulence factors and immunodominant antigens EsxA (CFP-10) and EsxB (ESAT-6) are expected to cause attenuation (22). The impacts of insertions in this region are summarized in Fig. 3 but are also available in Table S3 (“RD regions” tab) and Fig. S5. Insertions in genes encoding the structural components of the apparatus (*eccB1*, *eccCa1*, *eccCb1*, and *eccD1*) were significantly attenuated according to the criteria (adjusted *P* value cutoff of ≤0.05 and a log_2_ fold change of −1.5). Insertions in *eccA1*, which also codes for a structural component of the apparatus, were not attenuating despite good insertion saturation in this gene. This is supported by the work of others who have shown that deletion of *eccA1* in Mycobacterium marinum leads to only a partial secretion defect (23). There were no impacts seen due to insertions in accessory genes *espJ*, *espK*, and *espH.* The lack of attenuation seen in *espK* mutants is supported by other studies showing that this gene is dispensable for secretion through the apparatus and is not required for virulence of M. bovis in guinea pigs (24, 25). Insertions in *esxA* and *esxB* resulted in severe attenuation (log_2_ fold change of −6 to −7.5) but did not reach significance cutoffs (adjusted *P ≤ *0.05). This is likely to be due to the small number of TAs in these genes, which makes it challenging to measure mutant frequency, despite utilizing a pooled approach.

High levels of attenuation seen were in genes involved in the synthesis of the cell wall virulence lipids PDIMs (*ppsABCDE* and *mas* with log_2_ fold changes of −7 to 7.5 [Table S3, “Mycolipids” tab]). PDIM synthesis is well known to be required for the survival of M. tuberculosis and M. bovis in mice and guinea pigs (26, 27). MmpL7 is involved in PDIM transport, and there is evidence that it is phosphorylated by the serine-threonine kinase PknD (28). PknD-MmpL7 interactions are thought to be perturbed in M. bovis AF2122/97, as *pknD* is split into two coding sequences in the bovine pathogen by a frameshift mutation (29). The data presented here suggest that MmpL7 is required *in vivo* in cattle despite the frameshift mutation in *pknD.*

**FIG 4:**
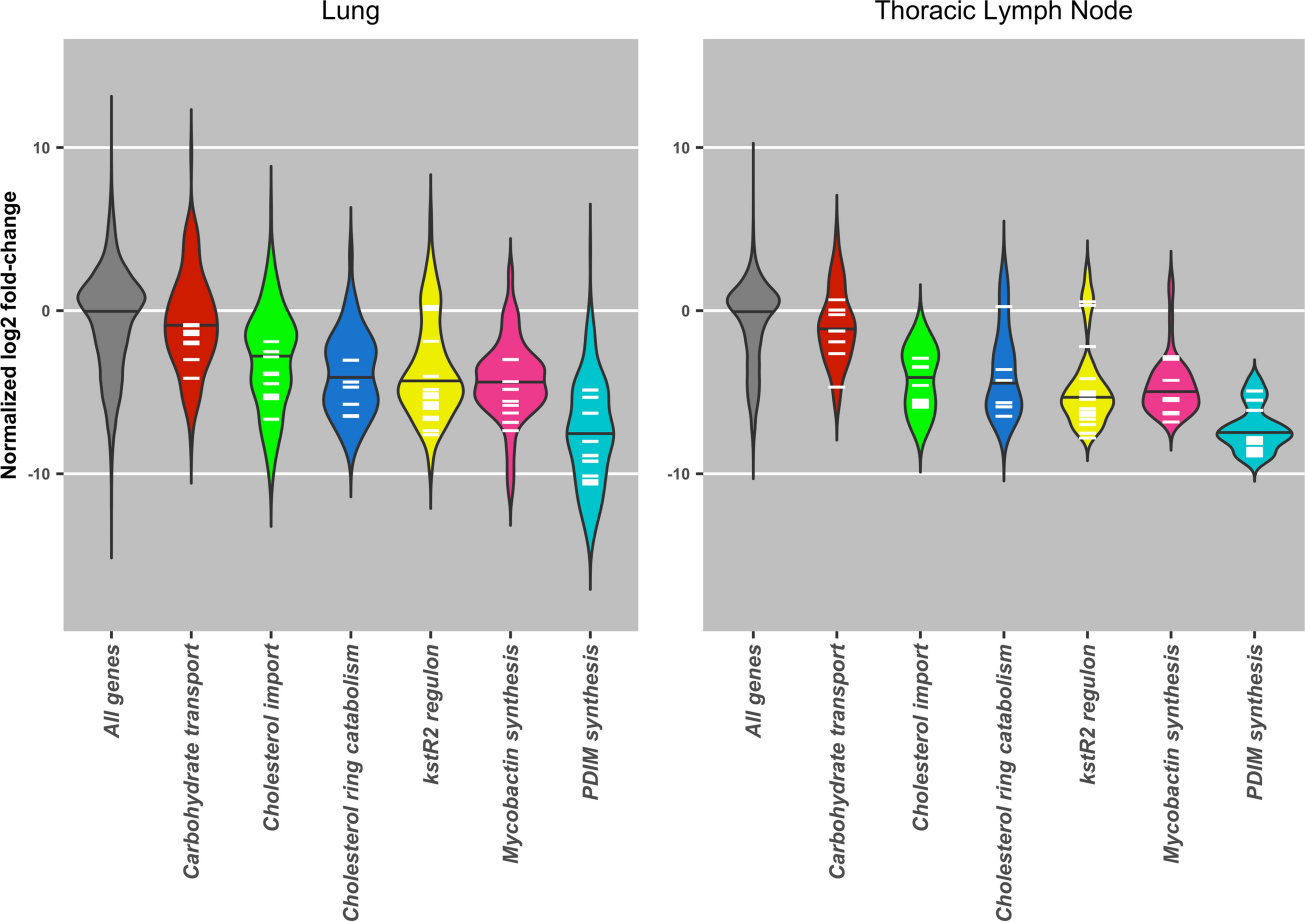
Violin plot of normalized log_2_ fold changes in gene insertions recovered from bovine lung or thoracic lymph node tissue samples in selected gene groups. Black bars indicate overall median of normalized log_2_ fold change among genes in grouping. White bars indicate mean log_2_ fold change for each gene in the group across all samples in either lung or lymph node tissue.

Iron restriction is thought to be a mechanism by which the host responds to mycobacterial infection, although different cellular compartments may be more restrictive than others (30). Insertion in many of the genes involved in mycobactin synthesis (*Mb2406-Mb2398c*, *mbtJ-mbtH)* were attenuating in cattle (Fig. 4; Table S3, “Mycobactin synthesis” tab). As mycobactin is required for the acquisition of iron, this confirms that, like other members of the MTBC, M. bovis needs to scavenge iron from the host for survival (10, 16).

**FIG 5:**
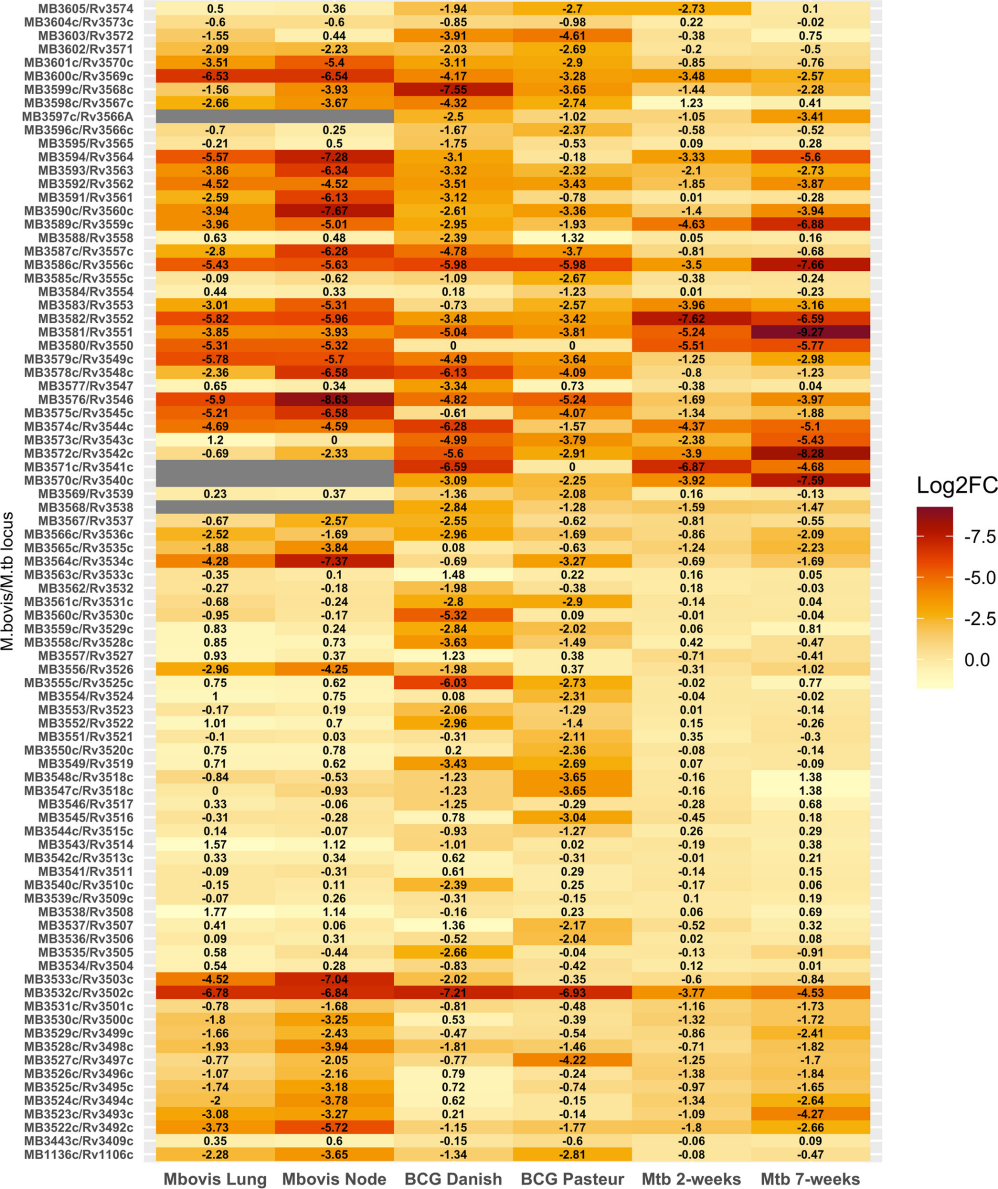
Comparison of reported log_2_ fold change in M. bovis, M. bovis BCG, and M. tuberculosis transposon insertion sequencing experiments for orthologous genes in the cholesterol catabolic pathway. Greatest attenuation (most negative log_2_ fold change) is colored darkest red. Studies used for comparison include Mendum et al. (24) and Bellerose et al. (9). Gray bars represent genes for which there is no information, as they were either essential (ES) or had a growth disadvantage (GD) in the input library or had less than 5 insertions in any TA site in any sample (input and all output).

The role of cholesterol catabolism in M. tuberculosis is well documented, and it is required for both energy generation and manipulation of the immune response (31–33). Cholesterol uptake is mediated by the Mce4 transporter coded by the *mce4* operon *Rv3492c-Rv3501c* (*Mb3522c-MB3531c*) (34, 35). It has been suggested that an alternative cholesterol acquisition pathway operates in M. bovis BCG Danish, as, unlike insertions in genes in the downstream catabolic pathway, insertions in the *mce4* operon do not result in attenuation in this strain (16). In contrast, our study shows that cholesterol transport via the Mce4 transporter is required in M. bovis AF2122/97 (Fig. 4; Table S3, “mce4 operons” tab). Interestingly, the significance cutoff (adjusted *P* ≤ 0.05) was only reached in the pooled lymph node samples, but it is difficult to say whether this indicates the requirement for Mce4 only occurs in the lymph nodes or if this is due to stochastic effects. The lymph nodes are the site of the engagement with the adaptive immune system and are the site of persistence for M. tuberculosis in nonhuman primates (36). The requirement for the Mce4 transporter corroborates work performed in M. tuberculosis, where Mce4 has been shown to be required for persistence in mice (8, 34). A comparison of the fitness impact on genes in the cholesterol catabolic pathway in M. bovis AF2122/97, M. bovis BCG Danish and Pasteur, and M. tuberculosis H37Rv is given in Fig. 5.

Early stages of cholesterol catabolism involve the oxidation of cholesterol to cholestenone, a reaction catalyzed by the 3β-hydroxysteroid dehydrogenase (*hsd*) encoded by *Rv1106c/Mb1136c. Rv1106c* is not required for the survival of M. tuberculosis in immunocompetent mice or guinea pigs, and this is thought to be due to the availability of other carbon sources, including glycolytic substrates, *in vivo* (8–10, 37). Insertions in *Mb1136c* in M. bovis AF2122/97 were attenuating (Table S3, “Cholesterol catabolism” tab), and this may be reflective of the inability of M. bovis AF2122/97 to utilize glycolytic substrates due to a disrupted pyruvate kinase (*pykA*) gene (38, 39). In a recent extended Tn screen utilizing diverse mouse genotypes, Tn insertions in *hsd* caused reduced fitness in a small panel of selected genotypes indicates there may be some host genetic component to the requirement for cholesterol oxidation by *hsd* (40). Given the potential for the use of host cholesterol metabolites, specifically cholestenone, as diagnostic biomarkers, this observation might have applications in the development of diagnostics (41).

### Genes that are differentially expressed between Mycobacterium bovis AF2122/97 and Mycobacterium tuberculosis *H37Rv*.

**FIG 6:**
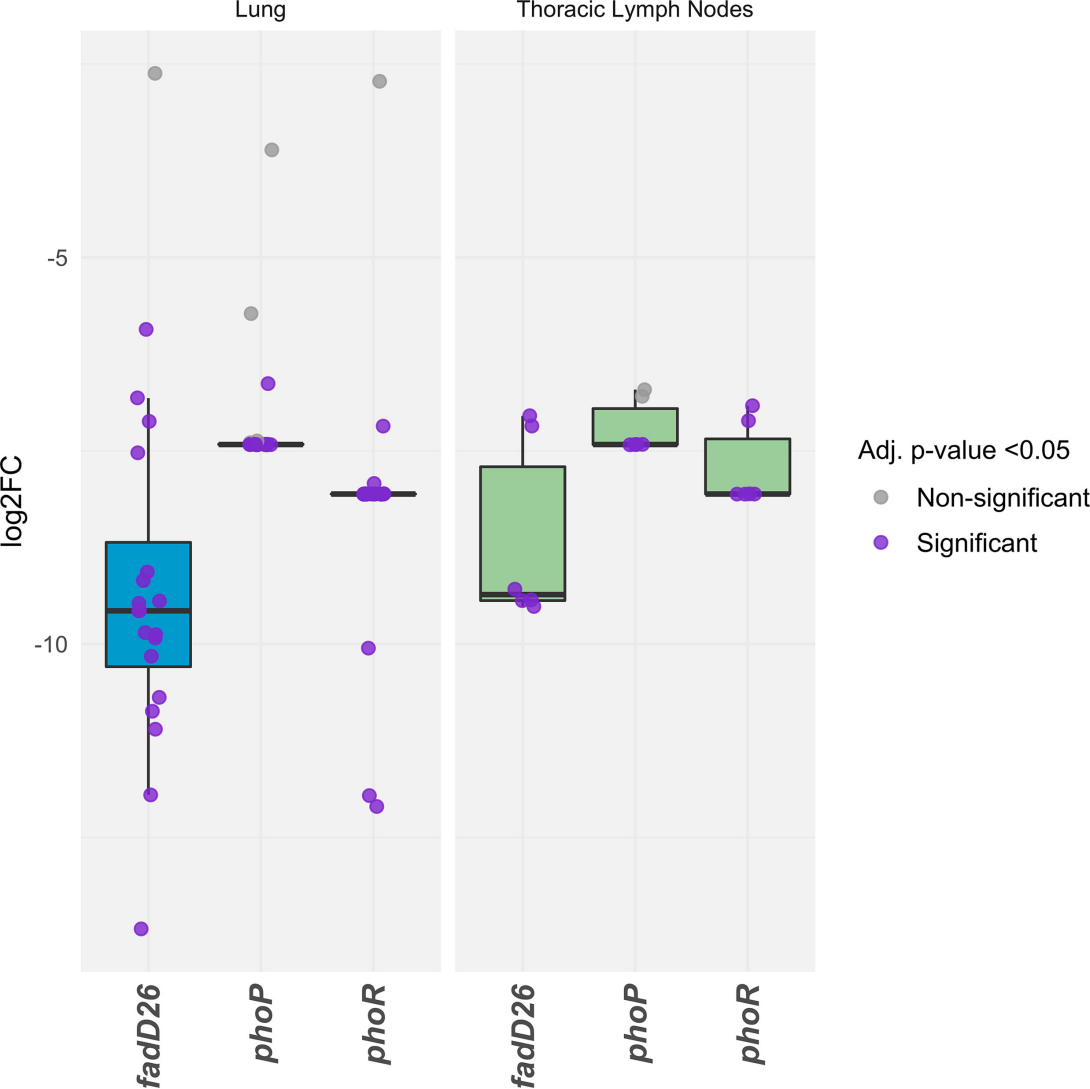
Fold changes caused by transposon insertions in *phoP*, *phoR*, and *fadD26* in the lungs and lymph nodes of infected cattle. Samples with adjusted *P* values (Benjamini-Hochberg FDR corrected) of <0.05 are indicated with purple points.

Several studies have identified key expression differences between M. bovis AF2122/97 and M. tuberculosis *H37Rv* (29, 42, 43). We examined the data set for insights into the role of differentially expressed genes and transcriptional regulators during infection. One important regulatory system in M. tuberculosis H37Rv is the two-component regulatory system PhoPR, and deletions in the *phoPR* genes alongside *fadD26* are attenuating mutations in the live vaccine MTBVAC (44–46). Our data show that insertions in both *phoPR* and *fadD26* caused attenuation (Fig. 6 and Table S3, “*phoPR* regulon” and “Mycolipids” tabs). This reinforces the role of this system in virulence despite the presence of a single nucleotide polymorphism (SNP) in the sensor kinase *phoR* that impacts signaling through the system in M. bovis AF2122/97 (44). However, care should be taken when using the data set to make inferences of the genetic requirements in field strains. For instance, *fadD26* contains nonsynonymous SNPs in global M. bovis collections (20). Signal potentiation via *phoR* is required for secretion of ESAT-6 through the ESX-1 secretory system, and M. bovis AF2122/97 is known to have compensatory mutations elsewhere in the genome, e.g., in the *espACD* operon that restores ESAT-6 secretion in the face of a deficient signaling system (44, 47). Our data also show that Tn insertions in *espA*, *espB*, and *espC* (required for ESAT-6 secretion) and in *mprA*, a transcriptional regulator of that operon (48), caused attenuation, emphasizing the relevance of ESAT-6 as a virulence factor.

Studies comparing differences in expression during *in vitro* growth between M. bovis AF2122/97 and M. tuberculosis H37Rv show that genes involved in sulfolipid (SL-1) biosynthesis are expressed at lower levels in M. bovis AF2122/97 than M. tuberculosis H37Rv (29, 42). Interestingly, insertions in genes involved in SL-1 biosynthesis (*Mb3850* to *Mb3856*) were not attenuating *in vivo* (Table S3, “Mycolipids” tab), reinforcing the lack of importance of SL-1 for M. bovis AF2122/97 *in vivo*, at least at the stages of infection studied here.

One of the most highly attenuated insertions occurred in *Mb0222/Rv0216*. This gene has been shown to be highly (>10-fold) overexpressed in M. bovis AF2122/97 compared with M. tuberculosis H37Rv, but the physiological function of this gene is not currently known. The secreted antigens MPB70 and MPB83, encoded by *Mb2900* and *Mb2898*, are also overexpressed in M. bovis AF2122/97 and play a role in host-specific immune responses; however, insertions in these genes did not cause attenuation *in vivo* in our data set (49).

### Novel attenuated mutations.

We identified 80 genes that were required for survival of M. bovis AF2122/97 in cattle that had not been previously described as being essential *in vivo* through transposon mutagenesis screens of M. tuberculosis in standard laboratory mouse models (8–10) (see Table S3, “Significant gene” tab). Insertions in some of these genes have been shown to cause attenuation in standard mouse models in M. tuberculosis through the use of single mutants (50–53). While writing this publication, a large-scale Tn-seq study that utilized over 120 M. tuberculosis libraries and several diverse mouse genotypes was performed (the collaborative cross-mouse panel [54]). This study captured the genes required for survival under a greater variety of host microenvironments than those performed in the standard mouse models (40). In that study, a larger subset of “adaptive” virulence genes that are required in a small subset of mice were identified, including those genes that were required in immunodeficient mice. Interestingly, insertions in *hsd* were attenuating only in immunodeficient mice in this study. A direct comparison of our data set with the study by Smith et al. revealed that a further 15 genes were shown to be required in at least two mice strains, hence classified as “adaptive” virulence genes with specific host genetic components contributing to fitness. There remains a subset of 65 genes that are required for optimal fitness of M. bovis AF2122/97 during infection of cattle that have not been previously identified as required for survival of M. tuberculosis in any mice by using transposon mutagenesis screens.

**FIG 7:**
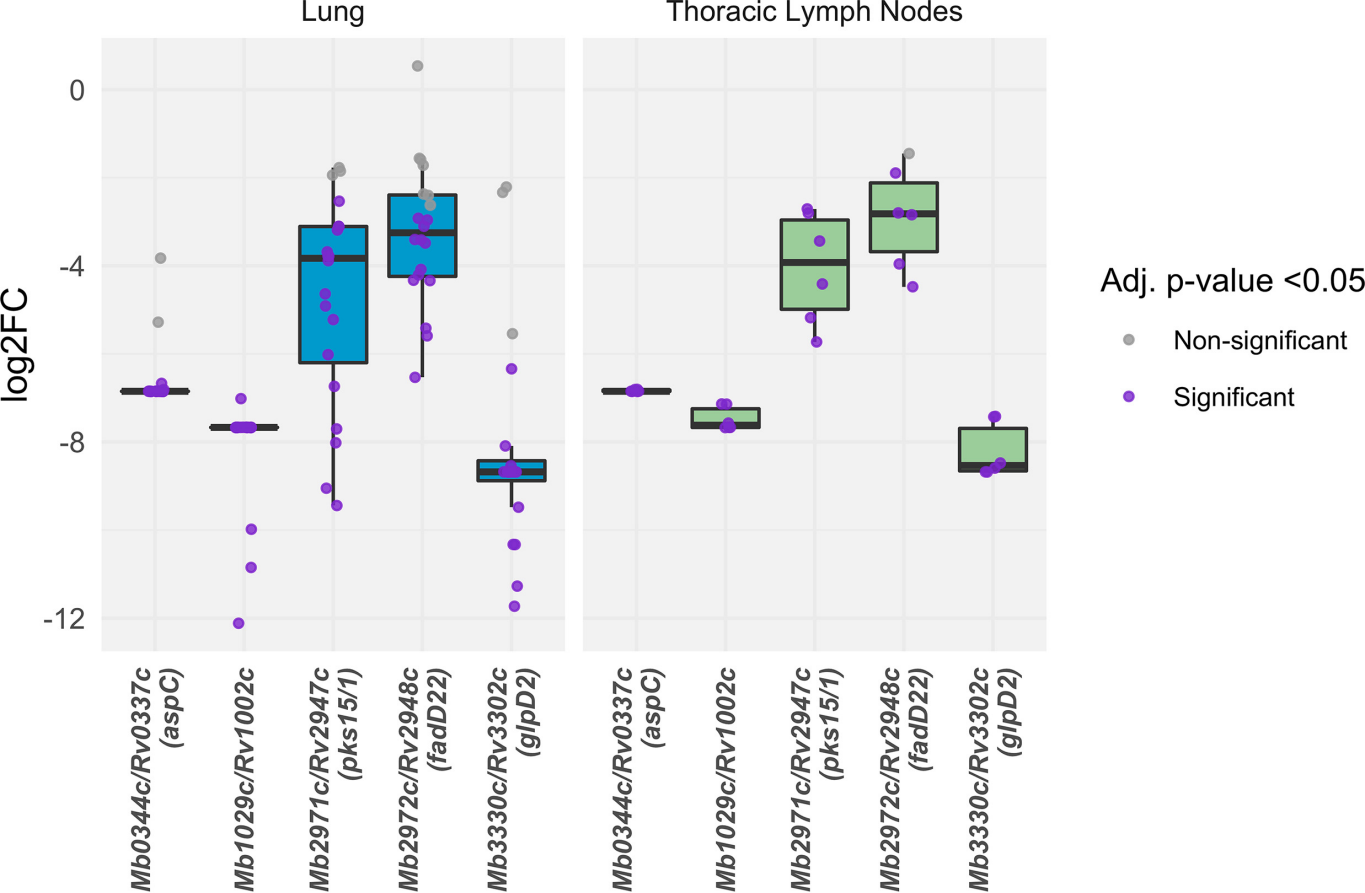
Fold changes caused by transposon insertions in *pks15/1*, *fadD22*, *Rv1002c*, *aspC*, and *glpD2* in the lungs and lymph nodes of infected cattle. Samples with adjusted *P* values (Benjamini-Hochberg FDR corrected) of <0.05 are indicated with purple points.

Genes required for phenolic glycolipid synthesis. Insertions in *Mb2971c/Rv2947c* (*pks15/1*) and in *Mb2972c/Rv2948c* (*fadD22*) were attenuating in M. bovis AF2122/97 (Fig. 7), but these genes are not required *in vivo* in M. tuberculosis H37Rv, including in the extended panel of mouse genotypes (8–10, 40). Both *pks15/1* and *fadD22* are involved in the early stages of synthesis of phenolic glycolipids (PGLs) and are involved in virulence (55). The requirement for these genes in M. bovis AF2122/97 but not in M. tuberculosis H37Rv is consistent with the observation that Tn-seq studies in M. tuberculosis are often carried out using lineage 4 strains (H37Rv and CDC1551) that harbor a frameshift mutation in the *pks15/1* gene, which renders them unable to synthesize PGLs. This removes the requirement for these genes *in vivo* in lineage 4 strains of M. tuberculosis*. pks15/1* has been previously reported to be required for survival of an M. bovis isolated in New Zealand in a guinea pig model of infection (56).

Genes that are involved in post-translational modifications such as glycosylation were attenuating in M. bovis AF2122/97 but not required *in vivo* in M. tuberculosis H37Rv*. Rv1002c* is thought to add mannose groups to secreted proteins, and overexpression of this protein in M. smegmatis was recently shown to enhance survival *in vivo* and inhibit proinflammatory cytokine production (57). The substrates of the protein mannosyltransferase are thought to be several secreted lipoproteins, including LpqW, which is involved in the insertion of the virulence lipid LAM at the mycobacterial cell surface (57, 58).

Finally, insertions in *aspC* and *glpD2* were attenuating in M. bovis AF2122/97 but not required *in vivo* in M. tuberculosis H37Rv. An examination of the *in vitro* essentiality literature showed that both of these genes are essential *in vitro* in M. tuberculosis H37Rv when grown on standard media but not in M. bovis AF2122/97 (11, 12, 19, 59). Information regarding *aspC* and *glpD2* from Tn-seq approaches is likely to be lacking in M. tuberculosis H37Rv because Tn mutants in these genes will not be represented in the input pool. The absence of insertion mutants in these genes in the most recent large-scale M. tuberculosis H37Rv Tn-seq study supports this (40). *aspC* (*MB0344c*/*Rv0337c*) is an aspartate aminotransferase involved in the utilization of amino acids (aspartate) as a nitrogen source (60). This provides evidence that M. bovis utilizes aspartate *in vivo*, as is observed in M. tuberculosis (61)*. glpD2* (*Mb3303c*/*Rv3302c*) is a membrane-bound glycerol-phosphate dehydrogenase. In Escherichia coli, *glpD2* is an essential enzyme, functioning at the central junction of respiration, glycolysis, and phospholipid biosynthesis, and catalyzes the oxidation of dihydroxyacetone phosphate (DHAP) from glycerol-3-phosphate, resulting in the donation of electrons to the electron transport chain (62). Its essentiality *in vitro* in M. tuberculosis H37Rv might be explained by the usage of glycerol during *in vitro* growth in this species. The contribution of the membrane-bound *glpD2* to the donation of electrons in the electron transport chain has been suggested but not yet explored in the MTBC (63). Given the interest in the electron transport chain as a chemotherapeutic target in M. tuberculosis, the data presented here suggest that inhibition of *glpD2* might be a fruitful approach in the development of new drugs for the treatment of TB in humans (64). The role of this gene in M. bovis AF2122/97 *in vivo* is perhaps surprising given the disruptions in glycerol phosphate uptake and pathways that phosphorylate glycerol in M. bovis AF2122/97 (65). However, M. tuberculosis is thought to engage in catabolism of membrane-derived glycerophospholipids, which may be an alternative potential source of glycerol-3-phosphate in members of the complex (66).

In this study, we have identified the genes required for survival in M. bovis AF2122/97 in cattle. The data set correlates well with preexisting knowledge. However, in addition to known requirements, we have uncovered novel virulence factors that had not previously been described in members of the complex. In this way, we both corroborate and expand our current knowledge of tuberculosis.

## MATERIALS AND METHODS

### Bacterial strains and culture methods.

M. bovis strain AF2122/97 was maintained on modified Middlebrook 7H11 (BD Difco) medium (65). Liquid cultures of M. bovis AF2122/97 were grown in Middlebrook 7H9 media (BD Difco) containing 75 mM sodium pyruvate, 0.05% (vol/vol), Tween 80, and 10% Middlebrook albumin-dextrose-catalase (ADC) (BBL BD Biosciences). Kanamycin at 25 μg/mL was used for selection where appropriate.

### Generation of input transposon mutant library and preparation of the inoculum.

Transposon libraries in M. bovis AF2122/97 were generated as previously described using the MycomarT7 phagemid system as per Majumdar et al. with modifications (18). Approximately 66,000 kanamycin-resistant transductants were scraped and homogenized in 7H9 medium and stored frozen at −80°C in 1-mL aliquots. CFU counting was performed on the homogenized culture to inform inoculum dosage.

### Cattle infection.

Experiments were carried out according to the UK Animal (Scientific Procedures) Act 1986 under project license PPL70/7737. Ethical permission was obtained from the APHA Animal Welfare Ethical Review Body (AWERB) (UK Home Office PCD number 70/6905). All animal infections were carried out within the APHA large animal biocontainment level 3 facility. Twenty-four Holstein-Friesian crosses of 6 months of age were sourced from an officially TB-free herd. An infectious dose of 7 × 10^4^ CFU was targeted for the “input” library, allowing each mutant to be represented in the library ~2.5-fold. Retrospective counting of the inoculum revealed the actual inoculum for infection contained 4 × 10^4^ CFU. The inoculum was delivered endobronchially in 2 mL of 7H9 medium.

### Infection monitoring with the IGRA.

Blood was collected by jugular venipuncture from animals on the day of the infectious challenge and 2 weeks after infection. Heparinized whole blood (250 μL) was incubated with purified protein derivative (PPD) from M. avium (PPD-A) or PPD from M. bovis (PPD-B) (Prionics), respectively, at 25 IU and 30 IU final. Pokeweed mitogen was used as the positive control at 10 μg/mL and a medium-only negative control. After 24 h of incubation in 5% (vol/vol) CO_2_, 95% humidity, 37°C atmosphere, blood was centrifuged (400 × *g* for 5 min); 120 μL of supernatant was removed and stored at −80°C for subsequent IFN-γ quantification using the Bovigam kit (Prionics) in accordance with the manufacturer’s instructions.

### Collection of tissues and gross pathology scores.

Six weeks after the initial infection, animals were subjected to postmortem examination. Initially, the experiment was designed with two time points, an early time point (6 weeks) and a later time point of 8 weeks. However, due to the unexpected high levels of pathology seen at the earlier time points, all animals were culled at 6 weeks. Gross pathology and evidence of TB-like granuloma lesions were scored using a modified methodology to that previously described in reference 67. Tissue from head and neck lymph nodes (from the right and left submandibular lymph nodes, the right and left medial retropharyngeal lymph nodes), thoracic lymph nodes (the right and left bronchial lymph nodes, the cranial tracheobronchial lymph nodes, and the cranial and caudal mediastinal lymph nodes), and from lung lesions was collected into sterile containers and frozen at −80°C until further processing. Frozen tissues were thawed and homogenized in phosphate-buffered saline (PBS) using a Seward Stomacher paddle blender.

### Recovery of the output transposon mutant library from tissues.

Tissue macerates collected from study animals were thawed at room temperature, diluted in PBS, and plated on modified 7H11 agar to determine bacterial loads. Colony counts were performed after 3 to 4 weeks of growth. For recovery of the library from tissue macerates, ~10^5^ to 10^6^ CFU were plated from lung lesions and thoracic lymph node lesions onto modified 7H11 media containing 25 μg/mL kanamycin. The colonies were plated over several 140-mm petri dishes to minimize competition between mutants. The colonies were harvested after 4 to 6 weeks of growth and genomic DNA extracted.

### Genomic DNA extraction.

Genomic DNA from the input and recovered libraries was isolated by an extended bead beating procedure with detergent-based lysis, phenol-chloroform DNA extraction, and precipitation as previously described (19). DNA quality was assessed by nanospectrometry (DeNovix) and gel electrophoresis and quantified by Qubit analysis using the broad-range assay kit (Thermo Scientific).

### Library preparation for transposon-directed insertion sequencing.

DNA (2 μg) was resuspended in 50 μL distilled water and sheared to approximately 550-bp fragments using an S220 focused ultrasonicator (Covaris) according to the manufacturer’s protocol. Fragmented DNA was repaired using NEBNext blunt-end repair kit (New England Biolabs) and purified using Monarch PCR cleanup kit (NEB). Blunted DNA was A-tailed using NEBNext dA-tailing kit (NEB) and column purified. Custom transposon sequencing adaptors (Table S4) were generated by heating an equimolar mix of Com_AdaptorPt1 primer and Com_AdaptorPt2 (P7+index) primers to 95°C for 5 min, followed by cooling by 1°C every 40 s to a final temperature of 4°C in a thermocycler. Adaptors were ligated to A-tailed library fragments using NEBNext quick ligase kit. Transposon-containing fragments were enriched by PCR using the ComP7 primer (10 μM) and an equimolar mix of primers P5-IR2a-d primer (10 μM) in a reaction with 50 ng of adaptor-ligated template and Phusion DNA polymerase (NEB) in a thermocycler with the following program: 98°C for 3 min; 4 cycles of 98°C for 20 s, 70°C 20 for s, and 72°C for 1 min; 20 cycles of 98°C for 20 s, 67°C for 20 s, and 72°C for 1 min; and 72°C for 3 min. Transposon-enriched libraries were subsequently purified with AMPure XP beads (Beckman), pooled, and further purified using AMPure XP beads.

### Data analysis.

Indexed libraries were combined, spiked with 20% PhiX, and sequenced on the Illumina HiSeq 3000 platform, using v2 chemistry, generating single-end reads of 250 bp. Raw FASTQ sequencing files were analyzed for quality and preprocessed using the TRANSIT TPP tool (68) set to default Sassetti protocol in order to remove transposon tags and adapter sequences and to map reads using BWA-MEM to TA sites to the M. bovis AF2122/97 genome (GenBank accession no. NC_002945). The TRANSIT tnseq_stats tool was run on each sample to assess insertion density, skew, kurtosis, and potential amplification bias.

The M. bovis AF2122/97 genome was scanned for the nonpermissive Himar1 transposon insertion motif (SGNTANC, where S is either G or C, and N is any base) as previously described (11). We identified 6,605 sites as nonpermissive (approximately 9% of total TA sites) and excluded them from resampling analysis. A custom annotation, prot-table for TRANSIT, was created from the M. bovis AF2122/97 annotation file (GenBank accession number LT708304, version LT708304.1). TRANSIT HMM was run on the input library using the default normalization (TTR) with locally estimated scatterplot smoothing (LOESS) correction for genomic position bias. Each TA site was assigned an essentiality state, and genes were assigned an essentiality call based on the assigned state of the TA sites within annotated gene boundaries.

Resampling between the input library and each of the output sample libraries was performed independently using the TRANSIT resampling algorithm and the complete prot-table. TTR normalization was used for 23 of the samples, and betageom normalization was used for the three samples with skew of greater than 50. The initial resampling output files were evaluated to identify genes with very few, or no, reads at any TA site within the gene boundaries in both the input library and output sample libraries. Genes with no read counts greater than 4 at any TA site, in any sample, and with a sum of all reads at any TA site across the 26 samples less than 55 were flagged. Essential and unchanged genes were removed from the prot-table prior to further evaluation. Resampling was further limited to protein-coding genes. Resampling was rerun for each sample using the edited prot-table and an edited TRANSIT resampling script to return the left-tail *P* value, as the data were expected to reflect attenuation. Resampling was performed in a separate analysis (“pooled”) with all of the sample insertion files as replicates using the edited TRANSIT resampling script and prot-table with betageom normalization. All *P* values were corrected for multiple testing with false-discovery rate (FDR) adjustment.

All analysis and plots were performed using R and R packages tidyverse and circlize (69–71). Orthologous TB genes were obtained from supplementary data files published by Malone et al. (29). All scripts, prot-tables, and insertion files are available at https://github.com/jenjane118/Mbovis_in-vivo_Tnseq and https://doi.org/10.5281/zenodo.6576716.

### Data availability.

Sequencing files (FASTQ) were deposited into BioProject under accession no. PRJNA816175.
